# Intravitreal Administration of HA-1077, a ROCK Inhibitor, Improves Retinal Function in a Mouse Model of Huntington Disease

**DOI:** 10.1371/journal.pone.0056026

**Published:** 2013-02-11

**Authors:** Mei Li, Douglas Yasumura, Aye Aye K. Ma, Michael T. Matthes, Haidong Yang, Gregory Nielson, Yong Huang, Francis C. Szoka, Matthew M. LaVail, Marc I. Diamond

**Affiliations:** 1 Department of Neurology, Washington University in St. Louis, St. Louis, Missouri, United States of America; 2 Beckman Vision Center, University of California San Francisco, San Francisco, California, United States of America; 3 UCSF School of Pharmacy, Department of Bioengineering and Therapeutic Sciences, University of California San Francisco, San Francisco, California, United States of America; 4 Department of Biopharmaceutical Sciences, University of California San Francisco, San Francisco, California, United States of America; National Center of Neurology and Psychiatry, Japan

## Abstract

Huntington disease (HD) is an inherited neurodegenerative disease that affects multiple brain regions. It is caused by an expanded polyglutamine tract in huntingtin (Htt). The development of therapies for HD and other neurodegenerative diseases has been hampered by multiple factors, including the lack of clear therapeutic targets, and the cost and complexity of testing lead compounds *in vivo*. The R6/2 HD mouse model is widely used for pre-clinical trials because of its progressive and robust neural dysfunction, which includes retinal degeneration. Profilin-1 is a Htt binding protein that inhibits Htt aggregation. Its binding to Htt is regulated by the rho-associated kinase (ROCK), which phosphorylates profilin at Ser-137. ROCK is thus a therapeutic target in HD. The ROCK inhibitor Y-27632 reduces Htt toxicity in fly and mouse models. Here we characterized the progressive retinopathy of R6/2 mice between 6–19 weeks of age to determine an optimal treatment window. We then tested a clinically approved ROCK inhibitor, HA-1077, administered intravitreally via liposome-mediated drug delivery. HA-1077 increased photopic and flicker ERG response amplitudes in R6/2 mice, but not in wild-type littermate controls. By targeting ROCK with a new inhibitor, and testing its effects in a novel *in vivo* model, these results validate the *in vivo* efficacy of a therapeutic candidate, and establish the feasibility of using the retina as a readout for CNS function in models of neurodegenerative disease.

## Introduction

Huntington disease (HD) is a devastating, incurable neurodegenerative condition, caused by CAG repeat expansion in the IT15 gene, which encodes an expanded polyglutamine tract in the target protein, huntingtin (Htt) [1]. One barrier to rapid development of therapies is the difficulty and expense in carrying out preclinical *in vivo* trials of candidate therapeutic compounds. This is due to multiple factors, especially inter-animal variability that requires large numbers of animals over relatively long study periods, and the challenge of drug delivery to the CNS. The R6/2 mouse [2] is widely used to study HD pathogenesis and test therapeutic leads. It expresses the first exon of the expanded Htt gene, which produces a highly neurotoxic and aggregation-prone protein. It exhibits rapid and uniform symptom onset at about 5–6 weeks of age, with death at about 14–19 weeks [2].

The R6/2 mouse has been reported previously to develop retinopathy [3], [4], but the histopathological descriptions have been only of the later-stage retinal phenotype at about 10 weeks of age, and no functional studies have been reported in this line. By contrast, the R6/1 line, which also expresses Htt exon 1, has later onset (13–21 weeks), and much later death (32–40 weeks) [2]. Its retinal pathology has been more thoroughly studied [2], [3], [5]. The retinal phenotype appears to be virtually identical in character in the two lines, differing only in time of onset and rate of degeneration. In the R6/1 line, measurement of retinal function by electroretinography (ERG) reveals decrements that precede histological changes [3], [5], suggesting that Htt toxicity manifests first with neural dysfunction. Since the rapid symptom evolution in R6/2 mice is much more favorable for assessing therapeutic intervention, we have carefully characterized the pathologic and physiologic features of retinal degeneration in this model. Our goal was to define a window of progressive dysfunction that would allow the efficient testing of an experimental compound with relatively small numbers of animals. We hypothesized that alterations in retinal function might serve as a robust readout for testing therapeutic candidates.

We have previously defined a signaling pathway involving the rho-associated kinase ROCK, whose inhibition by Y-27632 is beneficial in fly and mouse models of HD [6], [7]. ROCK directly phosphorylates its downstream target, profilin-1, a Htt binding protein. ROCK inhibition leads to dephosphorylation of profilin, which promotes Htt binding, and mediates the inhibitory effects of ROCK inhibitors on Htt aggregation [8]. Of note, a ROCK inhibitor HA-1077 (Fasudil) is in clinical use in Japan for subarachnoid hemorrhage [9], suggesting that it might represent a viable therapeutic lead for HD. To further test the therapeutic potential of ROCK inhibition, and to determine whether retinal dysfunction might serve as a viable readout for preclinical trials in general, we tested whether HA-1077 administration would improve retinal function in R6/2 mice.

## Materials and Methods

### Mouse husbandry

Ovarian transplanted wild-type female mice carrying R6/2 ovaries in B6/CBA background (Jackson Laboratory, Bar Harbor, ME) were mated with males of the same background. Male R6/2 mice were backcrossed with C57BL/6J females (Jackson Laboratory, Bar Harbor, ME) for more than seven generations. Genotyping and CAG repeat size was detected from tail DNA by PCR, as was the confirmation that the *rd1* gene (carried in some CBA substrains) was not present in the colony. Mice were maintained in a barrier animal facility at the University of California, San Francisco, housed five per cage with food and water available ad libidum, and kept on a 12-hr light/dark cycle. All studies and procedures were carried out under the approval of the Institutional Animal Care and Use Committees of University of California, San Francisco (Approval No. AN077961 and No. AN080876) in strict accordance with the recommendations in the Guide for the Care and Use of Laboratory Animals of the National Institutes of Health.

### Electroretinography

Mice were dark-adapted overnight and were anesthetized with an intramuscular administration of ketamine and xylazine while in dim red light. Corneas were anesthetized with 0.5% proparacaine, and pupils were dilated with 2.5% phenylephrine hydrochloride followed by 1.0% atropine. Bilateral, simultaneous full-field scotopic ERGs were elicited with 10-µsec flashes of white light, and responses were recorded using contact lens electrodes [10] with a UTAS-E 3000 Visual Electrodiagnostic System (LKC Technologies, Inc., Gaithersburg, MD) as described elsewhere [11]. The dark-adapted (scotopic) a- and b-wave response amplitudes and light-adapted (photopic) b-wave responses were then determined. In addition, we carried out flicker analysis of cone function after measuring light-adapted b-wave responses using a standard 20 Hz flicker stimulus at intensities of 0.4 and 0.9 log cd s/m^2^, averaging 50 responses.

### Morphological analysis using plastic sections

Mice were euthanized by CO_2_ inhalation. They were then immediately perfused with a fixative of 2% paraformaldehyde and 2.5% glutaraldehyde in phosphate buffered saline, and the eyes were subsequently removed. In some cases, eyes were enucleated and fixed by immersion in the same fixative. The eyes were then bisected along the vertical meridian and embedded in an Epon-Araldite mixture, with sections cut at 1 µm thickness and stained with toluidine blue as previously described [12]. Measurements of the outer nuclear layer (ONL) thickness were taken as an index of retinal degeneration, and were obtained from 54 locations around the retina, as described elsewhere [13]. Where the ONL was disrupted in focal regions, the ONL measurements were taken as close as possible to these disruptions. The number of cone nuclei at different ages was determined from the plastic sections by counting the cones in 10 microscopic fields of a 235-µm length of retina in a given section: 5 contiguous fields in the posterior retina superior to the optic nerve head and 5 fields in the inferior posterior retina as previously described [14], [15].

### Preparation of samples for immunohistochemical imaging

Mice were euthanized by CO_2_ inhalation and decapitated. Eyes were enucleated and fixed in 4% paraformaldehyde overnight, followed by a wash with phosphate-buffered saline. The cornea and lens were removed and the eye cups were embedded in OCT compound, or the whole retina was separated from the eye cup. Eye cups were cut on a cryostat (Leica 3000). The sections were air-dried for 20 min, and whole retina or dried sections were washed with 0.05% Tween 20 in phosphate- buffered saline, and then incubated with 5% goat serum and 0.1% Triton X-100 in phosphate buffered saline for 30 min at room temperature. Primary antibody incubation was performed at 4°C overnight for sections, or 48 hrs for whole retina. Samples were washed with 0.05% Tween 20 in phosphate-buffered saline for 1 hr for sections or overnight for whole retina. Goat anti-mouse IgG conjugated with Alexa 546 or Alexa 488 (1∶4000; Molecular Probes) was applied to the sections for 2 hrs at room temperature for sections, or 24 hrs overnight at 4°C for the whole retina in the dark. The sections and whole retina were washed for 1 hr and counterstained with 4′6′ diamidino-2-phenylindole (DAPI) (50 ng/ml) for 30 min, washed, and mounted with ProLong Gold anti-fade reagent (Molecular Probes). Fluorescence images were collected with a Nikon spectral laser confocal microscope C1s1.

### HA-1077 detection in mouse eyes

For initial studies of bioavailability of freely injected HA-1077, wild-type mice at 6 weeks of age were divided into two groups. One group of mice received intravitreal injection of 20 µM HA-1077 in both eyes, another group of mice received vehicle PBS in both eyes. Pairs of mice were sacrificed and whole eyes were enucleated and homogenized in H_2_O at 1 min, 12 min, 1 day, 3 days, 6 days and 9 days after injection. All mice that received PBS intravitreal injection were sacrificed at 9 days after injection, and eyes were enucleated and homogenized in H_2_O. Acetonitrile was added to the eye homogenate to precipitate protein, with chloroquine (100 ng/ml) as an internal standard (IS). HA-1077 concentration in the supernatant was measured by HPLC-tandem mass spectrometry (LC/MS/MS) as described previously [6]. To create reference standards, 14 known concentrations of HA-1077 (1.7–1700 ng/ml) were split into specimens of mice that received PBS intravitreal injection and carried through the extraction/purification procedure. The concentration of HA-1077 was measured by LC/MS/MS [6].

### Liposome preparation

Liposomes were prepared from PalmitoylOleoylphosphatidylcholine (POPC) and Cholesterol (Chol) with a molar ratio of 3∶2. Liposomes were treated with high vacuum overnight and rehydrated with 250 mM ammonium sulfate (pH 5.5). HA-1077 was loaded into the liposomes by the remote (active) loading method [16]. After loading, ammonium sulfate was dialyzed against a solution of 10 mM Hepes and 140 mM NaCl. The concentration of total lipid was 50 µmoles/ml. HA-1077 concentration inside the liposomes was determined by breaking down a small volume of the liposomes with 90% isopropyl alcohol 75 mM HCl and measuring drug concentration by Nanodrop chromatography with UV absorbance at 320 nM, comparing to reference standards.

## Results

### R6/2 mice show progressive retinopathy

R6/2 mice typically show the first motor deficits at 5–6 weeks and usually die by 12–19 weeks of age, depending on background strain and husbandry conditions [2] and the number of CAG repeats. We purchased R6/2 animals from the Jackson Laboratory (Bar Harbor, Maine), and maintained them on the C57BL/6J background. We used genetic screening by PCR to eliminate the risk of the recessive *rd8* mutation [17], which causes a slow retinopathy. We studied R6/2 mice at 6 to 19 weeks of age, and sacrificed animals at various time points to determine the characteristics of the retinopathy. The CAG repeat length ranged from 110–118, as determined by PCR.

**Figure 1:**
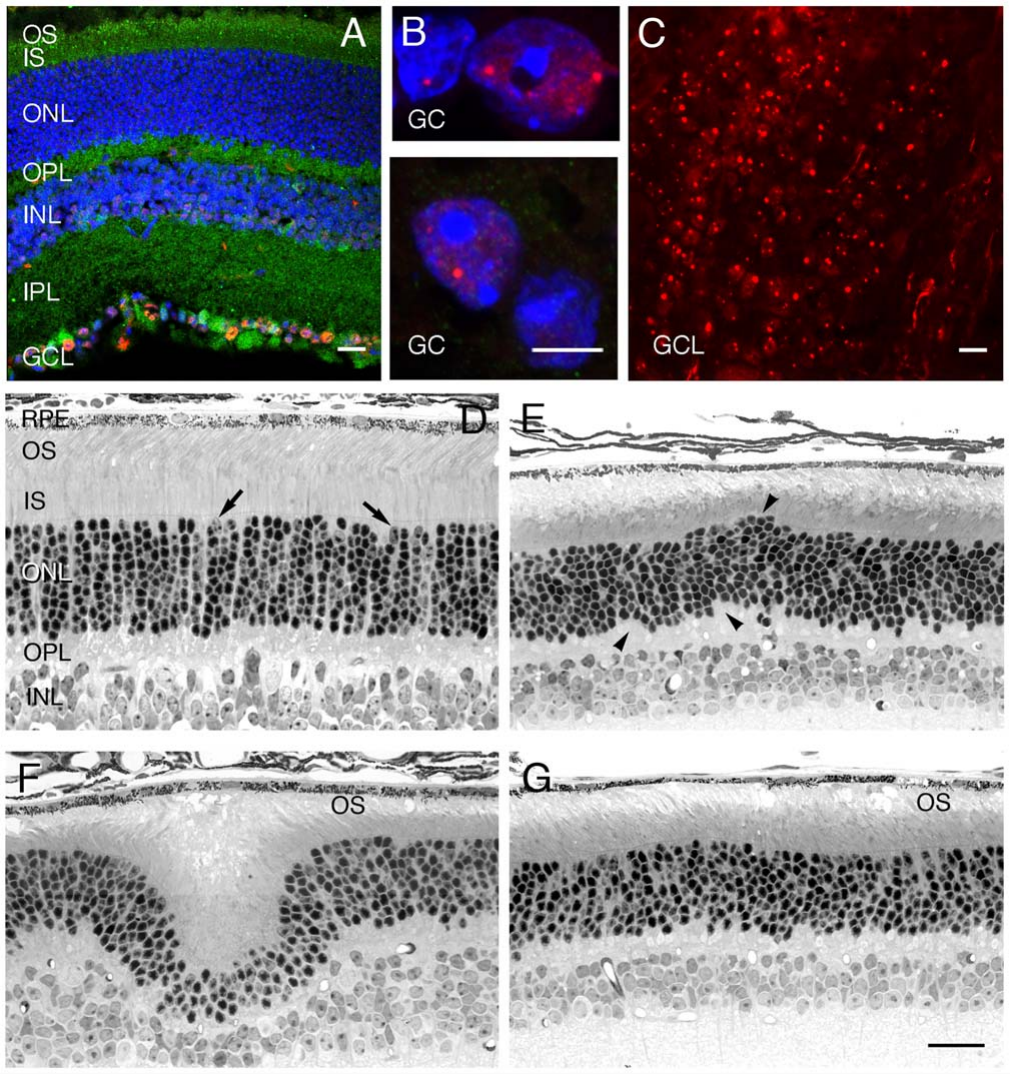
Expression of human huntingtin exon1 and morphological changes of R6/2 mouse retina. A,B: Frozen sections of 8-week-old R6/2 retina were co-immuno-stained with anti human huntingtin exon1 antibody EM48 (red) and anti phospho profilin1 antibody P3490 (green), and counterstained with DAPI (blue). Confocal microscopy was performed. A: EM48-positive inclusions can be seen in the retinal ganglion cell layer (GCL) and, B: in retinal ganglion cell (GC) nuclei at higher magnification with EM 48 positive (red) nuclear inclusions. C: 13-week-old R6/2 whole retina was stained with EM48 (red) and mounted on a microscope slide glass, then imaged by confocal microscopy. EM48-positive nuclear inclusions can be seen in many ganglion cells. D–G: plastic sections of the posterior retina of R6/2 mice of different ages. D: 8-week-old R6/2 retina that is indistinguishable from wild-type retinas of this age. Most photoreceptors in the outer nuclear layer (ONL) are rods, but cone nuclei are shown at the arrows. E: 11-week-old R6/2 retina showing small focal irregularities in the inner and outer borders of the ONL (arrowheads). F: 18-week-old R6/2 retina illustrating single, large fold in the ONL, giving the typical “wavy” appearance of the ONL in many regions that comprise 3–18% of the retinal length. G: 19-week-old R6/2 retina showing normal-appearing ONL, which is typical of the vast majority of retinal length at 18 to 19 weeks of age, but the photoreceptor outer segments (OS) are significantly shorter and more disrupted and vacuolated than normal. Scale bars: All except *B* = 25 µm; *B* = 10 µm. INL, inner nuclear layer; IPL, inner plexiform layer; IS, inner segments; OPL, outer plexiform layer; RPE, retinal pigment epithelium.

We used the anti-Htt antibody EM48 to stain for Htt accumulation. We observed that EM48-positive intranuclear inclusions first appeared in the retinal ganglion cells of R6/2 mice at approximately 7 weeks of age (Fig. 1*A–C*), extending to all 3 layers of the retina at approximately 11 weeks of age (data not shown). This roughly parallels the development of such inclusions elsewhere in the brain [2].

**Figure 2:**
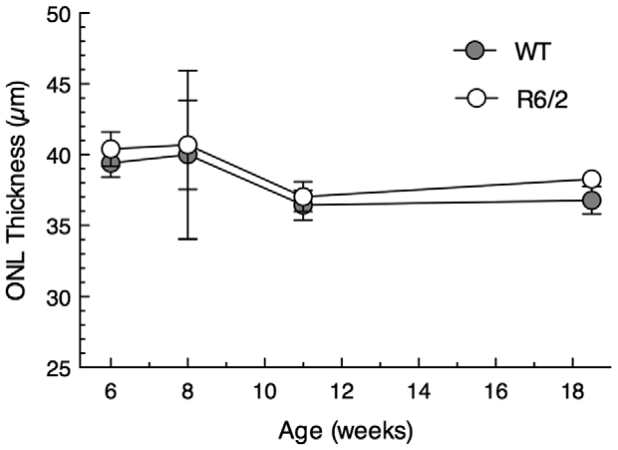
Outer nuclear layer thickness is similar in R6/2 and wild-type mice. The mean outer nuclear layer (ONL) of the retinas of R6/2 and wild-type (WT) mice are virtually identical at all ages studied. Mean ± SEM of 3–7 mice of each genotype at each age.

The R6/2 mice were indistinguishable from normal by high-resolution histology at 6–8 weeks of age (Fig. 1*D*). However, by 10–11 weeks, they began to have perturbations of the ONL (Fig. 1*E*), and thereafter developed the appearance thoroughly described by Helmlinger et al. [3] and Petrasch-Parwez et al. [4]. This included a focal irregular and ‘wavy’ ONL (Fig. 1*F*) predominantly located in the central-to-equatorial retina. While these undulations (retinal folds) were found in all of 10 mice examined at 18–19 weeks of age, they occupied a relatively small portion of the retinal length (7.4±4.4%, n = 10, range of 3–18%). Most of the ONL of these retinas had a normal appearance (Fig. 1*G*), but at all ages from 10–19 weeks, there was a disruption and shortening of the photoreceptor outer segments (Fig. 1*E–G*); some vacuoles in the interphotoreceptor space in the outer segment layer (Fig. 1*F–G*); some separation of the retina from retinal pigment epithelium (Fig. 1*D*); and displacement of some photoreceptor nuclei into the outer segment layer (not shown). The extent of the disorganization of the ONL became progressively more extensive in character and distribution by 18–19 weeks of age, but as reported previously for 10 weeks of age, the overall ONL did not become reduced in thickness at any age examined (Fig. 2), so relatively few rod nuclei were lost. Measurement of the ONL thickness is the most accurate and accepted measure of the relative number of photoreceptor nuclei [18]. It should be noted that the mouse ONL comprises about 97% rod nuclei and only 3% cone nuclei [19], so that any loss of cones would not be detected by ONL thickness measurements.

**Figure 3:**
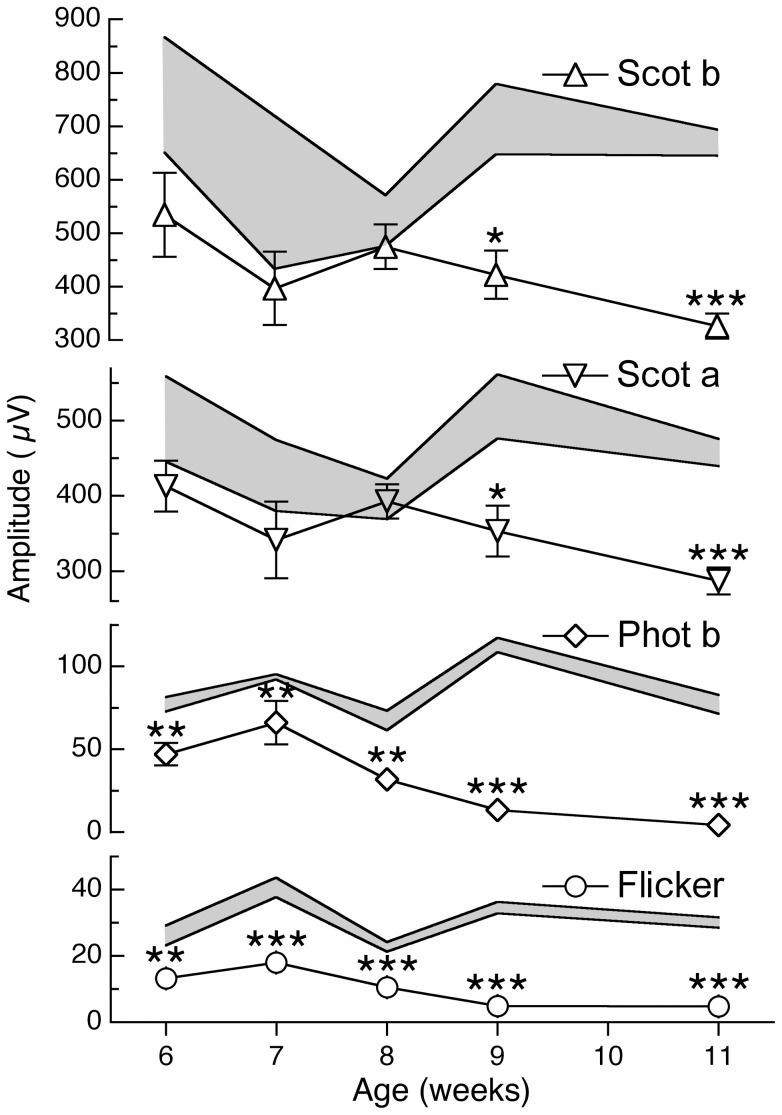
Cone-mediated ERG responses in R6/2 mice are lower than normal at all ages examined. ERG analysis of 6–19 week old R6/2 and wild-type mice at each age (see results). The light-adapted photopic b-wave (Phot b, open diamonds) and flicker (Flicker, open circles) cone-mediated ERG responses are significantly lower in amplitude than age-matched wild-type mice (range in shaded area) at all ages, beginning at 6 weeks of age. The scotopic a-wave (Scot a; open inverted triangles) and the scotopic b-wave (Scot b; open triangles) are significantly lower than wild-type controls beginning only at 9 weeks of age. *P<0.05; **P<0.005; ***P<5×10^−6^ by student t-test. Error bars represent S.E.M.

To define the characteristics of ERG changes over time, we studied multiple R6/2 and age-matched wild-type mice at various ages. We examined mice at 6 weeks (WT n = 8, R6/2 n = 8), 7 weeks (WT n = 6, R6/2 n = 9), 8 weeks (WT n = 15, R6/2 n = 11), 9 weeks (WT n = 13, R6/2 n = 13) and 11 weeks (WT n = 19, R6/2 n = 16) by ERG. Scotopic ERG responses, which predominantly measure rod (dark-adapted) function, remained relatively stable throughout the early portions of disease, and did not differ significantly from control values until 9 weeks of age. Both the scotopic a-wave and b-waves were lower than controls then and thereafter (Fig. 3). By contrast, photopic ERG response amplitudes were significantly lower than normal at all ages, beginning by at least 6 weeks of age. These primarily cone-mediated (light-adapted) responses were found with photopic and flicker ERG measurements (Fig. 3). Thus, the cone-mediated retinal deficit was evident at about the same age as the behavioral motor dysfunction, but before any detectable changes in retinal histology.

**Figure 4:**
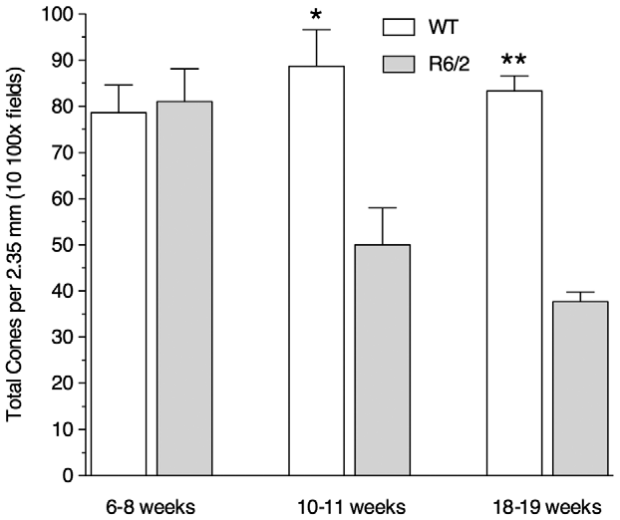
Cone photoreceptors are lost in R6/2 mice. Cone counts in 10 microscopic fields of 2–5 retinas at each age of R6/2 and wild-type (WT) mice. The average number of cones is similar in the R6/2 and WT mice at 6–8 weeks of age, but there is a reduction in the number of cones in R6/2 mice of about 44% at 10–11 weeks and of about 55% at 18–19 weeks of age. *p<0.05; **p<5×10^−9^ by student t-test. Error bars represent S.E.M.

The early decrease in cone function led us to examine the retinas for evidence of loss of cones. By counting cone nuclei in the retinas of R6/2 and wild-type retinas, we found that the number was virtually the same in both genotypes at 6–8 weeks of age. However, at 10–11 weeks and 18–19 weeks of age, the number of cones was reduced from normal by about 44% and 55%, respectively (Fig. 4). This method of counting cones has been effective and accurate in many studies of mouse retinal degeneration [13], [14], [20], [21].

The time window between 6–8 weeks of age represented a readily measured decline in photopic ERG responses at a time at which retinal cells were still intact. This suggested that this might be the optimal window at which to monitor effects of a putative disease modifier.

### Liposome-mediated drug delivery

The eye is a uniquely accessible CNS region to assess modifiers of pathology and neural dysfunction. It is possible readily to deliver experimental compounds without concern for bioavailability or systemic toxicity. We have previously described the neuroprotection afforded by Y-27632 in *Drosophila* [7] and in R6/2 mice with systemic administration [6]. HA-1077 (Fasudil) is a ROCK inhibitor approved for clinical use in Japan. It is employed to suppress vasospasm in subarachnoid hemorrhage [9], [22], [23]. We have previously observed that HA-1077 is more effective than Y-27632 in suppressing phosphorylation of profilin at Ser-137. It has a chemical structure distinct from Y-27632, and thus also serves as an additional validation of the putative target, ROCK. Preliminary experiments revealed that the half-life of HA-1077 injected directly into the eye was less than 24 hrs (data not shown), indicating that a more sustained release would be necessary. To improve the bioavailability of HA-1077, we packaged it into liposomes using palmitoyloleoylphosphatidylcholine (POPC) and cholesterol with remote loading approaches [24], [25], [26]. This method of drug delivery has been shown to provide sustained release within the intra-ocular space over several weeks [25], [26], [27].

### HA-1077 reduces profilin phosphorylation *in vivo*

**Figure 5:**
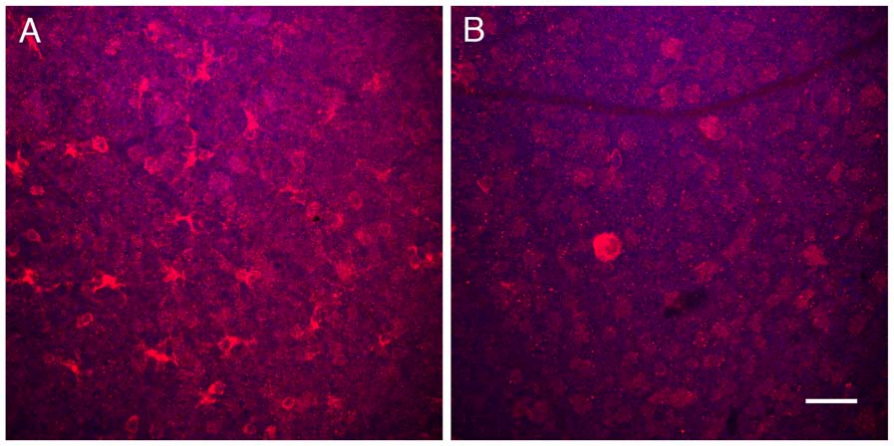
Intravitreal injection of liposomes loaded with HA-1077 decreases phospho-profilin 1 in mouse retina. One eye of 9-week-old wild-type mice (n = 4) was intravitreally injected with liposomes loaded with 40 µM HA-1077, and the other eye was intravitreally injected with empty liposomes. At 24 hours after injection, animals were sacrificed and whole retina cups were harvested, fixed and stained with anti-phospho profilin1 antibody P3490 (red), mounted on a microscope slide glass and imaged by confocal microscopy. A: The retina of eyes injected with empty liposomes shows many P3490-positive cells with irregularly shaped cytoplasm and processes. B: The retina of eyes injected with liposomes loaded with HA-1077 shows decreased staining density and few stained cells with round shaped cytoplasmic staining. Scale bar: 50 µm.

We have previously determined that Ser-137 of profilin is a direct target of ROCK, and we have developed a phospho-specific antibody to Ser-137 of profilin (P3490), that reports its phosphorylation [8]. We confirmed that liposome-mediated delivery of HA-1077 would produce the desired bioactive effect by administering the liposomes loaded with 40 µM HA-1077 to wild-type mice and measuring profilin phosphorylation in the retina. We injected 1 µl of liposomes loaded with HA-1077 intravitreally, and 1 µl of empty liposomes into the contralateral eye. After 24 hrs the animals were killed, and whole mounted retinas were stained with anti phospho-profilin antibody P-3490. Phospho-profilin levels were lower in HA-1077 injected eyes than that in empty liposome-injected eyes (Fig. 5). These experiments indicated that we were achieving bioactivity *in vivo* from the liposome-mediated delivery of HA-1077.

### HA-1077 improves cone-mediated ERG function

Next we tested whether chronic exposure of HA-1077 would attenuate the loss of photopic ERG signal we had documented in R6/2 mice (Fig. 3). We administered 1 µl of liposomes loaded with either 40 µM (WT n = 7, R6/2 n = 6) or 100 µM HA-1077 (WT n = 6, R6/2 n = 8) to 5-week-old mice. Since the total intravitreal space is approximately 3–4 µl, this was estimated to produce a final concentration of 10 µM or 25 µM, respectively. In each animal, the contralateral eye was injected with empty liposomes. The mice were allowed to recover and were maintained in their home cage environment for 2 weeks with standard 12-hr light/dark cycles. We then measured ERG amplitudes for both scotopic and photopic responses simultaneously in the two eyes. In each case, we compared the treated vs. the untreated eye, using a paired *t*-test. This paradigm is conventional for various kinds of therapeutic measures, because the ERG responses of the two eyes of individual normal and diseased retinas are highly symmetrical [11], [28], [29], [30]. Further, this method eliminates variables that can affect ERG response amplitudes between different animals, such as body temperature and level of anesthesia.

**Figure 6:**
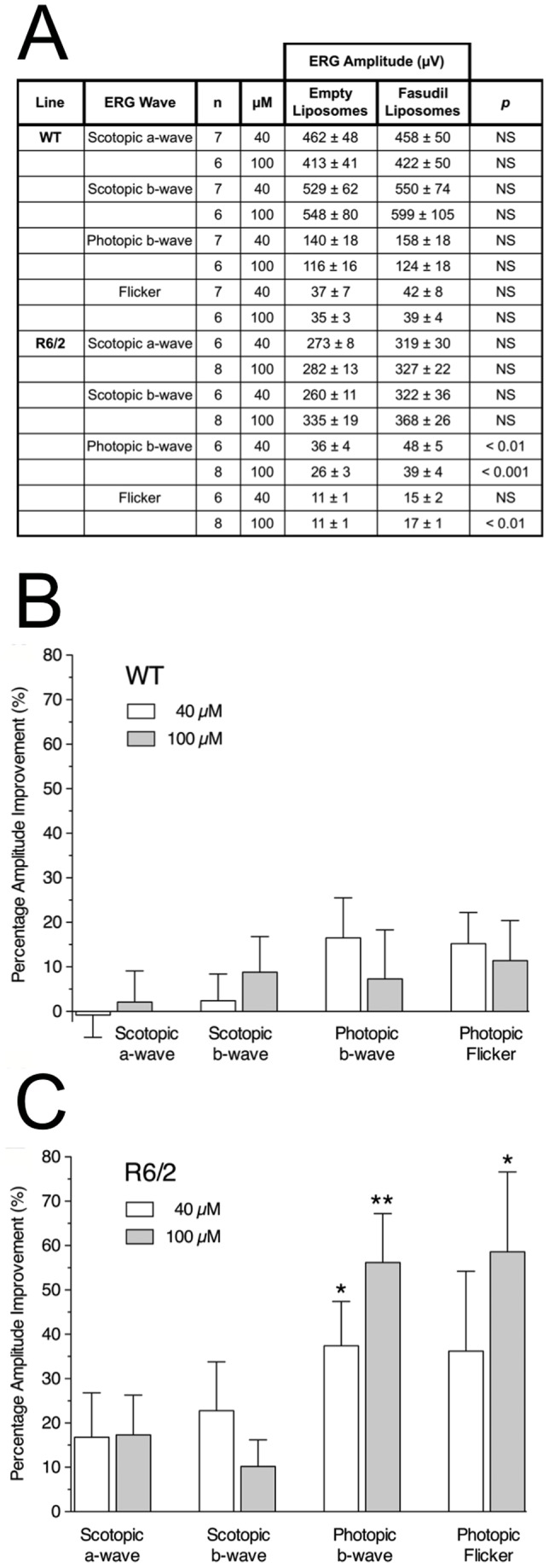
Intravitreal HA-1077 treatment improves cone-mediated ERG responses in R6/2 mice. Groups of R6/2 and WT littermate controls (n = 6–8 each) at 5 weeks of age were injected intravitreally in one eye with a single dose of liposomes loaded with either 40 µM or 100 µM HA-1077. The contralateral eye of each animal was injected with empty liposomes. ERG measurements were made 2 weeks later. A: Table of ERG responses. Amplitudes (µV) following intravitreal injection of empty or Fasudil-containing liposomes into the two eyes of wild-type and R6/2 mice. Values are indicated as Mean +/− S.E.M. B: In WT mice, HA-1077 had no significant effect on the ERG responses. C: In R6/2 mice, HA-1077 did not improve the rod-mediated scotopic responses, but both doses significantly improved the cone-mediated photopic b-wave and flicker ERG response amplitudes. Data are expressed as percentage improvement vs. the control liposome injected eye from each animal. *p<0.01; **p<0.001 by student t-test. Error bars represent S.E.M.

We examined ERG responses measured in the two eyes simultaneously in 8 wild-type and 5 R6/2 mice and found, as expected, that within an individual animal, the left and right eyes were relatively similar, with an average variance of less than 10% (data not shown). We reasoned that comparisons between the right and left eye in an individual animal were likely to be much more useful to test compounds than a comparison of different groups of treated animals, since intra-animal tests benefit from an internal control. HA-1077 treatment had no significant effect on ERG amplitudes in wild-type mice (Fig. 6A,B). However it significantly improved photopic and flicker ERG response amplitudes by ∼40% (40 µM dose) and ∼60% (100 µM dose) in R6/2 mice (Fig. 6A,C). Thus, HA-1077 improved cone-mediated responses before any histological signs of retinal degeneration. As expected for the relatively short treatment period, we did not observe any changes in Htt pathology in the treated eyes.

## Discussion

This study used ERG as a rapid, non-invasive measure of neuronal function in the R6/2 mouse model of HD to test a candidate therapeutic pathway. To begin, we established the characteristics of the progressive retinal pathology in R6/2 mice. We observed reduced photopic (cone-mediated) ERG responses at all ages examined, and a steady decline between 6 and 11 weeks of age. This was accompanied by loss of cone photoreceptors beginning at 10 weeks of age, at which time a progressive disorganization began in the outer retina. This roughly parallels decreases in rotarod performance in this line that we have previously observed in our laboratory [6]. By packaging HA-1077 within liposomes, we were able to deliver the compound on a sustained basis following a single intravitreal injection. We treated two cohorts of animals with different doses of HA-1077, using injection of the contralateral eye with empty liposomes as an internal control. This, coupled with the rapid ERG changes, allowed us to reduce the time and number of animals required to carry out the trial versus a conventional approach. HA-1077 treatment reduced the amount of phospho-profilin in the retina, indicating that we were hitting our therapeutic target. Chronic treatment (2 weeks) with a single injection of HA-1077 improved photopic ERG response amplitudes by 40–60%. These data indicate that ROCK inhibition reduces expanded Htt toxicity, possibly by inhibiting profilin phosphorylation.

### Cone-rod dystrophy in models of polyglutamine diseases

In addition to the R6/1 and R6/2 HD models, mouse models of spinocerebellar ataxia type 7 (SCA7) also feature retinal dystrophy [31], [32], [33], [34]. SCA7 is caused by a polyglutamine expansion in ataxin-7, rather than Htt. However, the cytopathology and sequence of structural and physiological changes are remarkably similar to those of R6/2 and R6/1, with the precise onset of symptoms dependent upon the particular line of SCA7 mice being studied. In each case, there is an initial identification of nuclear inclusions in the nuclear layers of the retina, an early loss of cone-mediated ERG responses that occurs at about the same time as motor symptoms and precedes the loss of rod-mediated ERG responses, and changes in the retinal ONL [3], [5], [31], [32], [33], [34], [35]. These ONL changes in all cases are characterized by a wavy ONL, but with little or no loss of layer thickness, and shortening of photoreceptor outer and inner segments. Where examined, changes in cone transduction molecules are reduced early [5], [31], [33], and there is a significant overlap in the changes in gene expression in the two disorders, with a huge down-regulation of cone-related genes in SCA7 mice [33] and in R6/2 mice [36]. The similarities of the cone-rod dystrophy in the two disorders imply common cytopathologic mechanisms in some polyglutamine diseases, as has been discussed [31], [35], [36]. In addition, the identification of specific cells affected first in the retina allows the possibility of targeting those cells therapeutically. A rod-derived cone viability factor has been identified [37], and approaches that exploit AAV vectors now exist to target cones [38].

### ROCK inhibition as a potential therapeutic mechanism for HD

ROCK has now been implicated by multiple studies in our laboratory as a potential HD modifier. Additional studies from other laboratories suggested that ROCK inhibition is potentially beneficial through effects on Htt degradation [39], [40]. Most recently ROCK and profilin were implicated as important components of the Htt interactome [41]. We have now studied ROCK inhibition in three animal models: Drosophila (oral administration of Y-27632) [7], R6/2 mice (oral administration of Y-27632) [6], and the current ocular study with R6/2 mice (intravitreal administration of HA-1077).

We have observed that two chemically distinct inhibitors of ROCK reduce Htt-mediated toxicity in the R6/2 mouse model. In our prior mouse study, with chronic oral administration of Y-27632, we estimated that we did not achieve fully therapeutic levels of Y-27632 in the brain [6]. In this study, on the other hand, we observed evidence of ROCK inhibition (reduction in phospho-profilin), which suggests that we achieved more effective bioactivity. We hypothesize that ROCK inhibition reduces the accumulation of toxic Htt oligomers, which are likely produced before the appearance of frank inclusions in this model. However, this study was not designed to measure effects on Htt protein aggregation *per se*, and the very small tissue volumes present in mouse retina make it very difficult to carry out biochemical studies to measure Htt protein solubility. Further, it remains to be determined whether changes in profilin phosphorylation alone can account for the therapeutic benefit we observed here, or whether changes in phosphorylation of other ROCK substrates play a critical role.

Prior to any studies in patients it will be important to test whether inhibition of this pathway will benefit a full-length Htt model, such has the BACHD mouse [42]. To our knowledge, HA-1077 has not been administered on a chronic oral basis to patients, and its CNS penetration outside the setting of acute brain injury may be limited. Thus, there could be significant barriers to introduction of this compound as a therapy for HD patients. Nonetheless, should it be tolerated on a chronic basis, ROCK inhibition could serve as a mechanism for an HD therapy.

### Retinal function as a facile readout of pathology

Testing of lead compounds in mice is expensive, time consuming and labor-intensive. Further, systemic administration of a compound must achieve adequate CNS levels, and inevitably leaves doubts about whether CNS neurons vs. peripheral tissues might be responsible for any improved behavior outcome. By contrast, it has been suggested that the retinal dystrophy in mutant mice might serve as a facile model for therapy in polyglutamine diseases [3], [34]. Here we have demonstrated the feasibility of using retinal physiology as a robust readout of a therapeutic effect *in vivo*. The retina is one part of the CNS that is readily accessible to chemical and genetic treatment. It is also uniquely amenable to accurately monitor neuronal physiology non-invasively via ERG. Although not used here, fundoscopy and optical coherence tomography (OCT) can also be employed to image neural anatomy *in situ*. Because any therapeutic trial can be conducted using the contralateral eye as an internal control, the number of animals needed to observe a statistically significant effect is vastly reduced. In this case, we have used the R6/2 mouse, which over-expresses an N-terminal exon 1 fragment of the Htt protein. Our studies here suggest that it should be possible to model toxicity *in vivo* for a variety of pathogenic proteins, simply by engineering animals that feature retina-specific expression. The use of the retina also allows modifications to be carried out on a very defined group of neurons, thus ensuring that specific CNS responses are linked directly to the targeted cells. Future work will help determine the predictive value of this system with other compounds that have demonstrated efficacy in standard transgenic models, and may ultimately speed preclinical testing.
